# Animal-Based Remedies as Complementary Medicines in the Semi-Arid Region of Northeastern Brazil

**DOI:** 10.1093/ecam/nep134

**Published:** 2011-02-13

**Authors:** Rômulo R. N. Alves, José A. A. Barbosa, Silene L. D. X. Santos, Wedson M. S. Souto, Raynner R. D. Barboza

**Affiliations:** ^1^Departamento de Biologia, Universidade Estadual da Paraíba, Avenida das Baraúnas, Campina Grande, Paraíba 58109-753, Brazil; ^2^Mestrado em Ciência e Tecnologia Ambiental, Universidade Estadual da Paraíba, Avenida das Baraúnas, Campina Grande, Paraíba 58109-753, Brazil; ^3^Pós-Graduação em Desenvolvimento e Meio Ambiente (PRODEMA), Universidade Estadual da Paraíba, Avenida das Baraúnas, Campina Grande, Paraíba 58109-753, Brazil

## Abstract

Animals (and their derived products) are essential ingredients in the preparation of many traditional remedies. Despite its prevalence in traditional medical practices worldwide, research on medicinal animals has often been neglected in comparison to medicinal plant research. This work documents the medicinal animals used by a rural community in the semi-arid region, inserted in Caatinga Biome, where 66 respondents provided information on animal species used as medicine, body parts used to prepare the remedies and illnesses to which the remedies were prescribed. We calculated the informant consensus factor to determine the consensus over which species are effective for particular ailments, as well as the species use value to determine the extent of utilization of each species. We recorded the use of 51 animal species as medicines, whose products were recommended for the treatment of 68 illnesses. The informant consensus in the use of many specific remedies is fairly high, giving an additional validity to this folk medicine. Eight species not previously reported as having medicinal use were recorded. The local medicinal fauna is largely based on wild animals, including some endangered species. Given a high proportion of medicinal animals observed in the study area, it is logical to conclude that any conservation strategy should include access to modern health care.

## 1. Introduction

The use of a complete range of natural resources including plants, animals and mineral inorganic components is a common practice in traditional medicine. Animals and products derived from their organs have constituted part of the inventory of medicinal substances used in various cultures since ancient times [1–4].

Despite its prevalence in traditional medical practices worldwide, research on medicinal animals has often been neglected in comparison to medicinal plant research [1, 5]. Emphasis was mostly put on medicinal plants because more different species as well as greater quantities are used than in the case for animals. Also, in many aspects plants are easier to collect, store and trade.

Recent publications have shown the importance of zootherapy in various socio-cultural environments around the world, and examples of the use of animal-derived remedies can currently be found in many urban, semi-urban and more remote localities in all parts of the world, particularly in developing countries [1, 6–10]. In Brazil, many species of animals have been used for medicinal purposes since colonial times, with widely disseminated therapeutic alternatives available throughout the country [1, 11–14]. Many people still use animal-derived medicines as an alternative or supplement to western health care [1, 15–17].

In Brazil, since the 1980s, various publications have shown the importance of zootherapy for traditional communities from distinct socio-cultural-environmental landscapes [1]. A recent review of the subject reported 290 different animal species being used in traditional folk medicine in Brazil [11]. This number is certainly underestimated since the amount of studies on the theme is very limited in number and locality. Most of what is known about medicinal animals comes from studies conducted in coastal areas and Amazon region (e.g., [15, 16, 18–21]). Comparatively, the least-acknowledged biomes are the Caatinga and Cerrado, two highly impacted ecosystems [22, 23]. The Caatinga (semi-arid) vegetation is a highly threatened biome covering a vast area in northeastern Brazil, and is the source of many little-studied natural resources [22, 24–28].

The use of animals for medicinal purposes is part of a body of traditional knowledge which is increasingly becoming more relevant to discussions on conservation biology, public health policies, sustainable management of natural resources, biological prospection and patents [5, 29–33]. In northeastern Brazil, especially in the semi-arid region, animals and plants are widely used in traditional medicine and play a significant role in healing practices [27]. Zootherapies form an integral part of the local culture, and information about animals and their uses are passed from generation to generation through oral folk lore. In this context, the present study contributes to the documentation of the animals utilized as medicines among a group of inhabitants of the rural area in municipal district of Queimadas, inserted in Caatinga Biome, located in the state of Paraiba, Brazil.

## 2. Methods

### 2.1. Study Area

**Figure 1:**
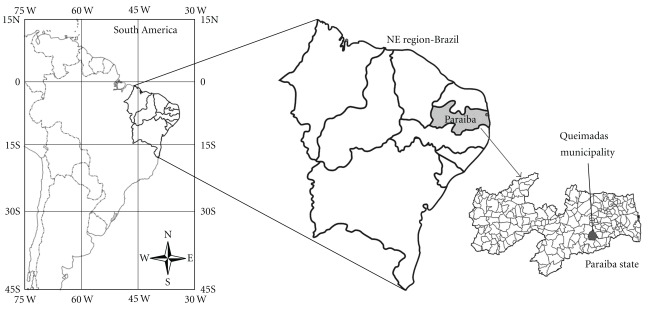
Map of study area, Municipality of Queimadas, Northeast Brazil.

The present work was carried out in community of Castanho de Baixo located in municipal district of Queimadas Paraíba State, Northeastern Brazil (Figure 1). Queimadas covers an area of 409 km^2^ and is situated in the Agreste mesorregion from state cited, at the approximate geographical coordinates 7°21′ S and 35°53′ W.

The climate is semi-arid and annual rainfall is between 400 and 800 mm, with wettest period from November to April. The local vegetation is composed of a spiny caducifolious vegetation characteristic of this semi-arid region (Caatinga biome).

In Queimadas the total population is *∼*39 000 of which, 56.2% live in the rural zone and 43.8% live in the city's urban area. The Human Development Index (HDI) is 0.595 (medium level development) [34]. In community studied (Castanho de Baixo), located in the rural zone, there are approximately 90 residences. The community of Queimada city is formed by typical Sertanejos people, descending from settlers families of the Agreste mesoregion of Paraíba.

About 130 years ago, the main existing forests in the mountains near the town provided excellent hunting, consequently leading the population to move there. These people began to populate the region, and since then still develop hunt activity in Castanho de Baixo area for food and commercialization. However, subsistence farming is the norm, primarily bean, corn, cassava, sweet potato, cattle, poultry, goats and sheep.

### 2.2. Procedures

Field research was conducted from September to November 2007 to February 2008. During the first contacts with the local population, we identified people with a specialized knowledge of medicinal animal use [15]. A specialist was defined as “a person recognized by the community as having deep knowledge about the use of animals in manufacturing remedies and in promoting cures”. Information on the use of animals in traditional medicines was collected through interviews with 66 persons (18 men and 48 women), mainly from the elderly populations, who still retain the major portion of traditional knowledge in their respective communities. Additional interviewees were chosen from referral. Prior informed consent was obtained for all interviews conducted. The ethical approval for the study was obtained from the Ethics committee of Paraiba University State.

Survey data were gathered through individual interviews [35] and included local name of the animal used as remedy; parts used as medicine; conditions treated with the remedy; preparation and usage; restrictions of use; adverse effects; spiritual aspects linked to the use; use of live or dead animals; how animals were obtained; storage conditions; collection sites; gear used to collect the animals; efficacy of the remedies; traditional uses of the remedies in the community; how knowledge was acquired by the interviewees; reliance on animal-based remedies; why the interviewee used animal-based remedies.

Species' vernacular names were recorded as quoted by interviewees. Zoological material was identified with the aid of specialists, through (i) examination of voucher specimens donated by the interviewees; (ii) photographs of the animals or their parts, taken during interviews; (iii) vernacular names, with the aid of taxonomists familiar with the study areas” fauna. Voucher specimens and/or photographs were deposited at the Department of Systematics and Ecology, Federal University of Paraíba.

### 2.3. Data Analysis

The reputed therapeutic effects and ailments treated were grouped into 16 categories based on the classification used by the Centro Brasileiro de Classificação de Doenças (Brazilian Centre for the Classification of Diseases) [36] (Table 1).

To estimate the level of agreement between interviewees over which animals to use for each category, we calculated the informant consensus factor (ICF), adapted from Heinrich et al. [37] that quantifies the variability of animals used for each treatment, and therefore the consensus between practitioners. This factor estimates the relationship between the “number of use-reports in each category (*n*ur) minus the number of taxa used (*n*t)” and the “number of use-reports in each category minus 1”. ICF is thus calculated using the following formula:

(1) $$\text{ICF}=\frac{nur-nt}{nur-1}.$$

The product of this factor ranges from 0 to 1. A value near zero indicates a high variation in the use of species, if animals are chosen randomly, or if informants do not exchange information about their use. Values near 1 indicate a high intra-cultural consensus.

The use-value (adapted from Phillips et al. [38]), a quantitative method demonstrating the relative importance of each species, was calculated as

(2) $$UV=\frac{U}{n},$$

where UV is the use-value of a species, *U* the number of citations per species and *n* is the number of informants.

Application of the use-value of each species is based objectively on the importance attributed by the informants and does not depend on the opinion of the researcher.

## 3. Results

**Figure 2:**
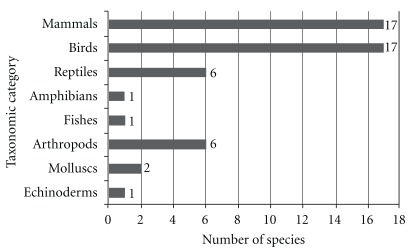
Number of animal species used as remedies per taxonomic category at the Municipality of Queimadas, Northeast Brazil.

**Figure 3:**
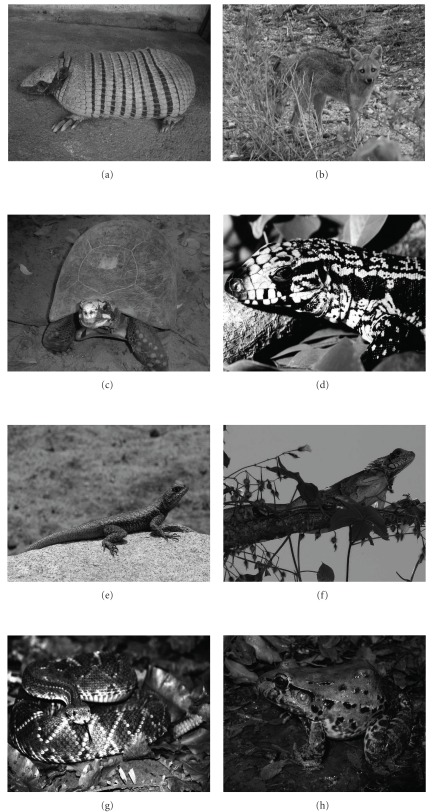
Examples of animals used as medicine in Northeast Brazil. (a) *Euphractus sexcinctus* (Photo: Wedson Souto), (b) *Cerdocyon thous* (Photo: Hélder Araújo), (c) *Chelonoidis carbonaria* (Photo: Rômulo Alves), (d) *Tupinambis merianae* (Photo: Yuri Lima), (e) *Tropidurus hispidus* (Photo: Washington Vieira), (f) *Iguana iguana* (Photo: Washington Vieira), (g) *Crotalus durissus* (Photo: Yuri Lima) and (h) *Leptodactylus vastus* (Photo: Claúdio Sampaio).

The study documented 51 medicinal animals (42 vertebrates and 9 invertebrates). The reported species were distributed among 42 zoological families. Birds and mammals (both with 17 species) and reptiles and arthropods (both with six species) were best represented in terms of the number of species (Figure 2). Examples of animals used as medicine are shown in Figure 3.

The most commonly mentioned species were *Gallus gallus domesticus—*domestic chicken and *Tupinambis merianae*—lizard (*n* = 59), *Ovis aries*—domestic goat (*n* = 56), *Atta cephalotes*—ant (*n* = 47), *Crotalus durissus*—rattlesnake (*n* = 46), *Euphractus sexcinctus*—six-banded armadillo (*n* = 43), *Iguana iguana*—common iguana (*n* = 43) and *Bos taurus*—domestic cattle (*n* = 42). The value use (VU) of zootherapeutic resources ranged from 0.03 to 0.893. The species which attained the highest use-value were *G. gallus domesticus* (0.893), *T. merianae* (0.893), *O. aries* (0.848), *C. thous* (0.742), *C. durissus* (0.696), *D. novemcinctus, I. iguana* (0.651) and *B. taurus* (0.636) (Table 2).

Although 85% medicinal animals were reported in previous studies carried in Brazil, we identified eight species not previously reported prescribed for treating a total of 18 illnesses: *Iphigenia brasiliensis*, *Molossus molossus, Buteogallus urubutinga*, *Megalobulimus oblongus*, *Protonectarina sylveirae, Netta erythrophthalma*, *Columba picazuro* and *Acromyrmex landolti*.

The local medicinal fauna was largely based on wild animals. Nevertheless, some domestic animal species are also used to produce materials for traditional medicine. These included the domestic cow (*Bos taurus*), domestic goat (*Capra hircus*), sheep (*Ovis aries*), dog (*Canis lupus familiaris*), ass (*Equus asinus*), horse (*Equus caballus*), domestic cat (*Felis catus*), pig (*Sus scrofa*), turkey (*Meleagris gallopavo*), helmeted guineafowl (*Numida meleagris*), “Codorna” (*Coturnix coturnix*), domestic chicken (*Gallus gallus*), Indian peafowl (*Pavo cristatus*) and Greater rhea (*Rhea americana*).

Interviewees quoted the following animal byproducts used as remedies: flesh, bone, cartilage, skin, tail, fur, feather, tooth, nail, head, tongue, stomach, viscera, liver, bile, milk, fat, rattle (from rattlesnakes), spine, shell, abdomen and body secretions (see Table 2). Hard parts, such as teeth, nails, fish scales, bone and cartilage were generally sun dried, grated and crushed to powder, being then administered as tea or taken during meals. Fat, body secretion and oil were ingested or used as an ointment.

Zootherapeutic resources were used to cure about 68 ailments. As reflected in Table 3, the body system categories with the greatest number of species and treatment indications were respiratory system (27 species; 320 use-citations), the undefined illnesses (20 species; 126 use-citations) and the osteomuscular system and conjunctive tissue (9 species; 177 use-citations).

Table 3 summarizes the ICFs for the 16 ailment categories recorded, showing the different levels of cultural consensus. The highest ICF values were for digestive and nervous system (ICF = 1) and for diseases of circulatory system (0.97). We reported that *C. coturnix* (UV: 0.56) was the most frequently used species for mental and behavioral perturbations and *P. sylveirae* (0.515) for circulatory system. The fourth highest ICF value (0.95) was recorded for diseases of osteomuscular system and conjunctive tissue, which was most often treated using the fox (*C. thous*) (UV: 0.74) and *C. durissus* (UV: 0.69).

Over 75.5% of animal species were reported to cure more than one ailment. For instance, products of the domestic chicken (*G. gallus domesticus*) were used to treat at least 11 illnesses and *I. iguana* was used to treat at least nine illnesses (Table 2). On the other hand, different animal species were sometimes used to treat the same illness. For instance, products obtained from 18 different species were used to treat asthma.

Animals provided the raw materials for remedies prescribed clinically and are also used in the form of amulets and charms in magic, religious rituals and ceremonies. Some respondents associated the use and efficacy of some remedies to popular beliefs locally known as “simpatias”. As examples they mentioned that animals' parts were used as amulets against diseases and that the person receiving a given treatment should not know the source else the effect would cease. Examples of “simpatias” include the use of teeth of “Prea” (*Cavia aperea*) as an amulet to treat teething; scrubbing a child's knee on “Donkey” (*Equus asinus*) footprints in order to make the child “walk early”; and “rattlesnake” (*C. durissus*) rattle as amulet to avoid serpents bites.

## 4. Discussion

Our study revealed that 51 medicinal animals were being used in surveyed area, indicating very rich ethnomedical knowledge of the local area. Eight of the identified species have not previously been reported as having medicinal use.

The high use of vertebrates reported in our study is in line with other studies [1–3, 7, 14, 15, 39–46]. With regards to habitat type, nearly all animals recorded were from terrestrial habitats (49 species)—a reflection of principal habitat types found in the surveyed area, located in the semi-arid region. Similarly, Adeola [3] has shown that in Nigeria the utilization of wildlife was related to the ecological zone in which the people lived, and to the relative abundance of species in each zone. This finding demonstrates the importance of local biodiversity in furnishing folk medicines, in agreement with Alves and Rosa [16] who observed that faunal composition, accessibility and availability directly influence the types of zootherapeutic items used in any given region.

The most of the medicinal animals were native to the semi-arid region, with the exception of *Oreaster reticulatus*, *Gadus morhua, Trichechus inunguis* and *Iphigenia brasiliensis*. These species were found at public markets in Campina Grande city, situated 22 km from the surveyed area, where marine species are traded [46]. The use of marine and estuarine species in the middle of the semi-arid region can be explained by the existence of established trade routes for medicinal animals throughout the north and northeast of Brazil [9, 15, 24, 40, 42, 46–50]. As pointed out by Alves et al. [46], the trade routes of medicinal animals traverse not only municipalities, but also Brazilian states.

Different parts of a single species provided the raw materials to prepare different remedies, which were prescribed to treat various diseases. The possibility of using various remedies for the same ailment is popularly valued, as it renders an adaptation to the availability/accessibility of the animals possible [15]. On the other hand, different species were sometimes used to treat the same illness. This strategy is important because many Caatinga species have a marked seasonality [28].

Zootherapeutical products are mainly used for the treatment of respiratory system diseases. A similar trend in relation to medicinal animals was found in cities of north and northeast of Brazil, where the two most frequently quoted categories of use referred to gastrointestinal and respiratory diseases [15, 16, 24, 40, 46].

Animals provide the raw materials for remedies used to treat physical and/or spiritual diseases. The use of some zootherapeutical resources is associated with popular beliefs [1, 2, 15–17, 24, 46]. Those links should be taken into consideration when interpreting results of field surveys and when designing public health programs for communities where traditional medicine is used. In some cases, integrative approaches encompassing an understanding of traditional cultural views and insights concerning the cause, dissemination and treatment of a disease might be required to effectively treat it [15].

Some of the medicinal animals that are used by the local people in present study find mention in ancient medicinal literature in Brazil. Examples of species used in Brazil since colonial times are: *I. iguana*, *C. durissus*, *Coragyps atratus* and *Bos taurus* [1, 42, 51]. This verification corroborates what Almeida [51] described as the “high capability of reproduction of zootherapeutic practices in Brazil”. The persistent use of animal-based medicines suggests that substances of therapeutic value not yet known by science may be present.

Despite their importance, analyses of the therapeutic use of animals and animal parts have been neglected, when compared with plants. However, within complementary and alternative medicine (CAM), zootherapy has been explored from the viewpoint of evidence-based [52], because the literature appears “glutted” with products derived from plants [53]. Both sources of natural products provide extensive sources of new CAM approaches that may emerge as important for future applications, including compounds isolated from marine microorganisms and phytoplankton, green algae, brown algae, red algae, fungi and certain well-known marine and terrestrial animals: sponges, coelenterates, bryozoans, mollusks, tunicates echinoderms, earthworms and leeches [54, 55].

It is widely accepted that folk or traditional medicinal uses (ethnomedical information) of biological resources indicate the presence of a biologically active constituent (s). In other words, folk or traditional medicinal uses represent “leads” that could shortcut the discovery of modern medicines [15, 16]. According to McGirk [56], Brazilian scientists are studying a type of frog that is used to cure intestinal illnesses by members of the Yawanawa Indian tribes. Indeed, amphibians have provided compounds capable of being turned to therapeutic advantage. Peptides extracted from the scraped secretions of *Phyllomedusa bicolor*, for instance, are used in the treatment of depression, stroke, seizures and cognitive loss in ailments such as Alzheimer's disease [57]. Some of these compounds are important tools for biochemical research or as new leads for the development of anticancer or antiviral drugs [58]. Regarding fish, several compounds have been extracted and these are employed as remedies in the official medicine [59]. Finkl [60], for example, refers to *Eptatretus stoutii*, *Dasyatis sabina*, and *Taricha* sp. as sources of cardiac stimulants, antitumors and analgesic, respectively. Oily fish, like cod, herring, salmon and turbot, have a great medicinal value to human beings due to a polyunsaturated compound known as OMEGA-3. This substance helps the prevention of arthritis [61]. The presence of an anticoagulant system in the plasma of Atlantic salmon (*Salmo salar* L.) and rainbow trout (*Oncorhynchus mykiss* Walbaun) has been confirmed, which supports similarities with the protein C anticoagulant system in mammals [62]. Geckos, frogs and other various insects are used in many Asiatic Materia Medica; meloid (“blister”) beetles and leeches were listed for a long time in Western Pharmacopoeias and maggots has been recently listed in the US Pharmacopoeia [63, 64].

Clinical studies are lacking for most [5, 65]. However, in the absence of scientific knowledge, consensus among practitioners in the use of particular remedies for particular ailments and level of usage may indicate effectiveness of use [66]. This can potentially be followed up by clinical studies and aid in the development of pharmaceutical drugs [67]. Among the species quoted by interviewees in the present study, some have previously been tested and their therapeutic effects evaluated. Murari et al. [68] provided evidence showing that *P. cristatus* feather extract inhibited hyaluronidase and proteolytic enzyme activities caused by the venom of *Vipera russelii*, *Naja naja* and *Trimeresurus malabaricus*, demonstrating a mechanism by which it could neutralize venom toxicity. Bisset [69] showed that the analgesic substances in the venom of species belonging to the families Viperidae, Crotalidae and Elapidae are more potent than morphine. The analgesic properties of venoms of some snake species, including *C. durissus terrificus* venom, have been demonstrated in humans and in experimental animal models [70–74]. Honey produced by honeybees (*Apis mellifera*) has both bacteriostatic and bactericidal effect [75]. Park et al. [76] isolated milk proteins of lactoforicine type from *B. taurus* with activity against bacteria. Cow urine has also been found to increase phagocytosis by macrophages and thus is sought helpful in prevention and control of bacterial infections. Besides this, cow urine has antioxidant property which protects DNA damage due to mitomycin-C-induced chromosomal aberrations [77].

In Brazil, most of the medicinal animals used are collected from the wild [1, 14–18, 40, 46, 77, 78]. This same trend was observed in this study, where 28 (77.7%) of the species traded are sourced from the wild. Nevertheless, many domestic animal species are also used to produce materials for traditional medicine.

The common use of domestic species may result from the ease of obtaining those animals and/or decline in wild fauna populations due to overhunting and loss of habitat. Human activities such as slash and burn agriculture, goat and cattle raising and extensive subsistence hunting are thought to be causing severe environmental impoverishment and a loss of biodiversity in the Caatinga [23].

This study also identified nine species of medicinal animals on either the IUCN Red List of Threatened Species (http://www.iucnredlist.org/), CITES list (Convention on International Trade in Endangered Species of Wild Fauna and Flora (http://www.cites.org/eng/resources/species.html), Brazil's official list of endangered species [79] or the National List of species of aquatic invertebrates and fishes endangered, overexploited or threatened by exploitation [80].

Unscrupulous usage of animal products in traditional medicines has led to many undesirable consequences including illegal trafficking of animal products. Pocking of animals for their medicinally important parts has brought many of the wild species under the red data book, for a possibility of their extinction. Many genera and species of wild animals have been considered at the brim of extinction as a consequence of overexploitation either of their own or of their habitat [2, 29]. Anyinam [81] pointed out that, like the current spasm of plant and animal species extinction, the practitioners of ethnomedicine appear to be at a greater risk of extinction than even forests and other biomes. Environmental degradation affects users of traditional medicine both by limiting their access to the resources traditionally used and by diminishing the knowledge base in their community upon which traditional medicine is constructed.

The medicinal use of animals must be considered together with other anthropogenic pressures [1, 2]. Rapid reduction in natural resources as a consequence to the expanded urbanization, global warming and reduced natural habitat posed a considerable threat to the sustainability of traditional medicine. Being completely dependent upon natural resources like herbs, minerals and animal products, traditional medicine would possibly rank first in order of extinction of heritage if an alternative way is not considered well in time [29].

There is a need to increase our understanding of the biology and ecology of species commonly used as remedies to better assess the impacts of harvesting them (for medicinal or other purposes) on their wild populations. Medicinal species whose conservation status is in question should receive urgent attention and aspects such as habitat loss and alteration should be discussed in connection with present and future medicinal uses [1].

The idea of sustainability in traditional medicine can well be traced through different cultures and societies with different notions [82–84]. Zootherapeutic activity, if properly managed, can be compatible with an environmental conservation program in which the use of natural resources can and must occur in such a way that human needs and protection of biodiversity are guaranteed [15]. The therapeutic indications of wild animals and plants and domestic or cultivated species overlapped in many cases. This aspect opens the possibility of, where suitable, replacing the use of threatened species with others in traditional medicine recipes [1, 2]. Using domestic species does not represent a threat to the ecosystem and on top of that the use of such species may lead to large-scale use of natural products, as long as their efficacy is confirmed.

Besides being influenced by cultural aspects, the relations between humans and biodiversity in the form of zootherapeutic practices are conditioned by the social and economic relations between humans themselves. We would suggest further studies on these traditional remedies to confirm the presence of any bioactive compounds and also include this traditional knowledge into the strategies of conservation and management of faunistic resources for sustainable use.

Using animal products as components of bioprospecting has implications for medicines, the environment, economy, public health and culture [5, 85–89]. There is a great necessity to educate the local population and healers to adopt conservation measures as necessary, so that over collection of such species will not lead to their extinction in their territory, which signifies the loss of their source medicinal material. Given the high proportion of medicinal animals observed in the study area, it is logical to conclude that any conservation strategy would improve health care, particularly for rural communities with limited access to modern health facilities.

## Figures and Tables

**Table 1:** Categories of diseases treated with zootherapeutic remedies in surveyed area (Queimadas, Paraiba State), according to the Brazilian Centre for the Classification of Diseases.

Categories	Diseases and illnesses mentioned by respondents	Total
1	Teething, inflammation, hoarseness, cracks in the sole of the feet, general pain, to assist children who take longer than usual to start walking, children that speak with lateness, itching, problems of navel, “hard nerve” and healing of umbilical cord of newborn baby	11
2	Erysipelas, oral mycosis, athlete's foot, measles, rubella, chickenpox, tuberculosis, warts, whooping cough and mumps, moniliasis	11
3	Asthma, bronchitis, effusion, catarrh, cough, flu, sore throat, sinusitis and tonsillitis	9
4	Poor digestion	1
5	Burns, wounds, muscle strain, luxation, distension and sprains	6
6	Arthritis, arthrosis, backache, osteoporosis and rheumatism	5
7	Hemorrhage, cardiac problems, thrombosis and cerebral hemorrhage	4
8	Menstrual problems and urinary infection	2
9	Alcoholism, snakes bite, insect bite, to suck a splinter out of skin or flesh, swelling and bleeding	6
10	Boils, skin spots and acne,	3
11	Problems in nerves	1
12	Tumors	1
13	Deafness, earache	2
14	Diabetes, weakness and malnutrition	3
15	Sexual impotence	1
16	Conjunctivitis and pterygium	2

Total		68

(1) undefined diseases; (2) some infections and parasitic diseases; (3) respiratory system; (4) digestive system; (5) injuries, poisoning and other consequences of external causes; (6) osteomuscular system and conjunctive tissue; (7) circulatory system; (8) urinogenital system; (9) external causes of morbidity and mortality; (10) skin and subcutaneous tissue; (11) nervous system; (12) Symptoms, signs and abnormal findings from medical and laboratorial examination, not categorized in other part or section; (13) ear (middle and inner ear) and mastoid apophysis; (14) diseases of the endocrine glands, metabolism and nutrition; (15) mental and behavioral perturbations; (16) ophthalmological diseases.

**Table 2:** Animal taxa recorded as having medicinal properties.

Family/species/local name	Number of mentions	Relative importance (UV)	Part used and way of administration	Disease (or illness)
Echinoderms				
Oreasteridae				
*Oreaster reticulatus* (Linnaeus, 1758)-Starfish, “estrela-do-mar”	7	0.106	Whole animal (3, 5)	Asthma
Mollusks				
Megalobulimidae				
*Megalobulimus oblongus* (MÏller, 1774)-clam	4	0.06	Whole animal (5)	Asthma
Donacidae				
*Iphigenia brasiliensis* (*Lamarck*, 1818)-“concha-do-mar”	3	0.045	Shell (8)	Teething
Arthropods				
Apidae				
*Apis mellifera* (Linnaeus, 1758) “abelha” (Africanized honey bee)	22	0.333	Honey (4, 18)	Bronchitis, “catarrh in the chest”, coughs, influenza, sore throat, sinusitis, tonsillitis, hoarseness, tuberculosis and whooping cough
Blattidae				
*Periplaneta americana* (Linnaeus, 1758)-American cockroach, “barata”	14	0.212	Offal (9)	Earache
			Whole animal (3)	Asthma
Formicidae				
*Acromyrmex landolti* (Emery, 1980)-“saúva”	11	0.166	Whole animal (5)	Asthma
*Atta cephalotes*-“tanajura”	47	0.712	Abdomen (4)	Sore throat, “catarrh in the chest”, cough and hoarseness
Termitidae				
*Nasutitermes macrocephalus* (Silvestri, 1903)-Termite, “cupim”	39	0.590	Whole animal (16)	Bronchitis, “catarrh in the chest” coughs, influenza, sore throat, sinusitis, tonsillitis and hoarseness
Vespidae				
*Protonectarina sylveirae* (Saussure, 1854)-“marimbondo-preto” “marimbondo-mosquito”	34	0.515	Nest (19, 5)	Mumps, hemorrhage, blooding and menstrual problems
Fishes				
Gadidae				
*Gadus morhua*, Linnaeus, 1758^VU^-Atlantic cod “Bacalhau”	3	0.045	Fat (2, 6, 4)	Backache and rheumatism
Amphibians				
Hylidae				
*Leptodactylus vastus,* Lutz, 1930-“Jia”	18	0.272	Fat (4), meat (1, 12)	Sore throat
Reptiles				
Chelidae				
*Mesoclemmys* tuberculata (Luederwaldt, 1926)-Tuberculate toadhead turtle, “cágado”, “cágado-d'água”	38	0.575	Fat (2, 6, 17)	Column pain backache, rheumatism, swell, furuncles and tumors
Crotalidae				
*Crotalus durissus* (Linnaeus, 1758)^DD/III^-Neotropical rattlesnake, “cascavel”	46	0.696	Fat (2, 6, 4)	Rheumatism, pains in general, backache, and inflammation
			“Maracá” (rattle) (8)	Snake bite
Iguanidae				
*Iguana iguana* (Linnaeus, 1758)^DD/II^-Common iguana, “camaleão”	43	0.651	Fat (2, 6, 4, 9)	Rheumatism, pains in general, column pain, sore throat, earache, arthritis, osteoarthritis, furuncles and tumors
			Bones (3, 14)	Rheumatism, arthritis and osteoarthritis
Teiidae				
*Tupinambis merianae* (Duméril & Bibron, 1839)^DD/II^ *-* Lizard,“teju”, “tejuaçú”	59	0.893	Fat (4, 9, 2, 6, 17)	Sore throat, earache, “catarrh in the chest”, coughs, influenza, hoarseness, tumor and swelling
Testudinidae				
*Chelonoidis carbonaria* (Spix, 1824)^DD/II^-Red-footed tortoise, “jabuti”	9	0.136	Fat (2, 6, 17)	Furuncles, tumors and pains in general
			Whole animal (11)	Asthma
Tropiduridae				
*Tropidurus hispidus* (Spix, 1825)-“lagartixa” (Lizard)	34	0.515	Offal (6)	Erysipelas, chilblain, warts, skin spots and cracks in the sole of the feet
			Tail (5)	Asthma
			Whole animal (1, 5, 6)	Asthma, chilblain, warts and skin spots
Birds				
Accipitridae				
*Buteogallus urubitinga* (J. F. Gmelin, 1788)-“gavião-cauã”	3	0.045	Bones (3, 14)	Backache, Column pain and rheumatism
Anatidae				
Anas platyrhynchos Linnaeus, 1758-“patos” (Domestic duck)	27	0.409	Eggs (4, 12)	Male impotence, and weakness
*Netta erythrophthalma* (Wied-Neuwied, 1833)-“paturi”	19	0.287	Eggs (4, 12)	Male impotence and weakness
Cathartidae				
*Coragyps atratus* (Bechstein, 1793)-“urubu” (Black vulture)	18	0.272	Liver and feather (3, 5, 15)	Asthma, alcoholism
			Whole animal (16)	Tuberculosis
Columbidae				
*Columba livia* (Gmelin, 1789)-“pombo” (Rock pigeon)	6	0.090	Meat (12)	Asthma
*Columba picazuro*, Temminck, 1813-“asa-branca”	5	0.075	Whole animal (1, 12)	Sore throat, tonsillitis, bronchitis and hoarseness
*Leptotila rufaxilla* (Richard & Bernard, 1792)-Gray-fronted dove, “juriti”	8	0.121	Gizzard (10)	Pterygium
Corvidae				
*Cyanocorax cyanopogon* (Wied, 1821)-White-naped jay, “cancão”	21	0.318	Whole animal (11)	Asthma
Falconidae				
*Herpetotheres cachinnans* (Linnaeus, 1758)^LR/II^-“acauã” (Laughing falcon)	3	0.045	Whole animal (1)	Sore throat, tonsillitis and hoarseness
Meleagrididae				
*Meleagris gallopavo* Linnaeus, 1758-turkey, “peru”	13	0.196	Fat (17, 2, 6)	Furuncles, tumors and cracks in the sole of the feet
			Feather (3)	Asthma
Numinidae				
*Numida meleagris* Linnaeus, 1758-Helmeted Guineafowl, “Guiné”	9	0.136	Fat (6, 17)	Tumors and furuncles
			Whole animal (1)	Pertussis
Phasianidae				
*Coturnix coturnix* (Linnaeus, 1758)-“codorna”	37	0.560	Eggs (4, 12)	Male impotence, urinary infection and weakness
*Gallus gallus domesticus* (Linnaeus, 1758)-Domestic chicken, “galinha”	59	0.893	Fat (4, 2, 6, 17, 9)	Bronchitis, “catarrh in the chest”, coughs, influenza, sore throat, sinusitis, tonsillitis, swelling, furuncles, tumors, and earache
			Gizzard (5)	Poor digestion
			Eggs (6)	Problems of navel, Healing of umbilical cord of newborn baby
			The whole animal (13)	It is long of the child to begin to speak to assist children who take longer than usual to start walking
*Pavo cristatus* Linnaeus, 1758-“pavão”, Indian peafowl	16	0.242	Featherses (3)	Asthma
Psitacidae				
*Amazona aestiva* (Linnaeus, 1758)-“papagaio”	5	0.075	Feces (5, 6)	Asthma, skin spots and acne
Rheidae				
*Rhea americana* (Linnaeus, 1758) ^LR/II^-Greater rhea, “ema”	4	0.060	Eggs (4, 12)	Weakness and malnutrition
			Fat (2, 6)	Pains in general
Tyrannidae				
*Fluvicola nengeta* (Linnaeus, 1766)-Masked water-tyrant, “lavandeira”	12	0.181	The whole animal (3)	Asthma
Mammalia				
Bovidae				
*Bos taurus* Linnaeus, 1758-“gado” (cattle) (cow)	42	0.636	Bone marrow (1, 12)	Problems in the nerves and weakness
			“Fel” (bile) (2, 6)	Suck a splinter out of skin or flesh
			Excretion urinary (2, 4)	Chilblain, diabetes and conjunctivitis
			Penis (3, 5)	Asthma
*Capra hircus* Linnaeus, 1758-“cabra”, “bode” (Domestic goat)	33	0.5	Milk (4, 12)	Weakness and malnutrition
			Hair (3)	Asthma
*Ovis aries* (Linnaeus, 1758)-“carneiro” (Sheep)	56	0.848	Suet (2)	Hard nerve, suck a splinter out of skin or flesh, cardiac problems, inflammation, sprains and swelling
Canidae				
*Canis lupus familiaris* (Linnaeus, 1758)-“cachorro” (Domestic dog)	19	0.287	Feces (5)	Measles and chicken pox
*Cerdocyon thous* (Linnaeus, 1766)^LR/II^- “raposa” (Fox)	49	0.742	Fat (2)	Arthritis, osteoarthritis, osteoporosis, rheumatism, column pain, sprain and swelling
Caviidae				
*Cavia aperea* Erxleben, 1777-“Preá”	16	0.242	Fat (2, 6)	Crack in the feet cracks in the sole of the feet,
			Teeth (8), head (1) and whole animal (1)	Teething
*Kerodon rupestris* (Wied-Neuwied, 1820)-“Mocó” (Rock cavy)	15	0.227	Fat (9)	Deafness
			Estomach (5)	Thrombosis and effusion
Dasypodidae				
*Dasypus novemcinctus*, (Linnaeus, 1758)-“tatu”	43	0.651	Tail (9)	Deafness and earache
Didelphidae				
*Didelphis albiventris* (Lund, 1840)-“timbú” (Common opossum)	8	0.121	Fat (2, 6)	Arthritis, osteoarthritis, osteoporosis, backache, rheumatism and sprains
Equidae				
*Equus asinus* Linnaeus, 1758-“jumento” (Asino)	37	0.560	Milk (4)	Weakness and malnutrition to assist children who take longer than usual to start walking
			“Trace” (footprints) (6)	
*Equus caballus* (Linnaeus, 1758)-“cavalo” (Horse)	7	0.106	Hair (7)	Warts
Felidae				
*Felis catus* Linnaeus, 1758, 1775-“gato” (Domestic cat)	4	0.06	The whole animal (6)	Rubella
Hominidae				
*Homo sapiens* Linnaeus, 1758-“gente” (People)	11	0.166	Excretion Urinary (2, 6, 4)	Itching, insect bite, conjunctivitis and diabetes
Mustelidae				
*Conepatus semistriatus* (Boddaert, 1785)-“tacaca” (Striped hog-nosed skunk)	11	0.166	Fat (2, 4)	Arthritis and osteoporosis
Suidae				
*Sus scrofa* (Linnaeus, 1758)-“porco” (Pig)	28	0.424	Fat (2, 6)	Crack in the feet, burns and wounds
			“Fel” (bile) (2, 6)	Suck a splinter out of skin or flesh
Trichechidae				
*Trichechus inunguis* (Nattrer, 1883)^VU/I^- “peixe-boi” (Amazonian manatee)	5	0.075	Fat (2, 4)	Wounds, inflammation, sprain, muscle strain, suck a splinter out of skin or flesh, arthritis, osteoarthritis, osteoporosis and rheumatism
Molossidae				
*Molossus molossus* (Pallas, 1766), Pallas' free-tailed bat (Bat)	2	0.03	The whole animal (3)	Asthma

(1) ingestion of the cooked broth; (2) ointment to be rubbed in the affected area; (3) tea of the toasted powder; (4) ingestion of the raw part; (5) tea; (6) to place on the affected area; (7) to use as cable; (8) to use as amulet; (9) introduced in the ear; (10) powder on the affected area; (11) to create as estimate animal; (12) ingestion of the cooked part; (13) introduced in the mouth; (14) powder ingested with food; (15) mixed with alcoholic drink and taken as drink; (16) mixed with sugar and taken as syrup; (17) mixed with plants to do cataplasm; (18) mixed with plants and taken as drink; (19) dissolved and used as cataplasm. IUCN Red List Categories: DD: Deficient Data, LR: Least Concern, VU: Vulnerable. Cites Appendix-I, II or III.

**Table 3:** ICF categorized by medicinal use for corporal ailment.

Ailment category	Species	All species (%)	Use citation	All use citations (%)	ICF
Undefined illnesses	20	37.73	126	8.09	0.848
Some infections and parasitic diseases	9	17.64	106	6.95	0.923
Respiratory system	25	49.01	299	19.61	0.919
Digestive system	1	1.88	38	2.44	1
Injuries, poisoning and other consequences of external causes	8	15.09	117	7.51	0.939
Osteomuscular system and conjunctive tissue	9	16.98	177	11.37	0.954
Circulatory system	2	3.77	49	3.14	0.979
Urinogenital system	2	3.77	6	0.38	0.800
External causes of morbidity and mortality	8	15.09	123	7.90	0.942
Skin and subcutaneous tissue	8	15.09	119	7.64	0.940
Nervous system	1	1.88	8	0.51	1
Symptoms, signs, and abnormal findings from medical and laboratorial examination, not categorized in other part or section	7	13.20	81	5.20	0.925
Ear (middle and inner ear) and mastoid apophysis	6	11.76	69	4.52	0.926
Diseases of the endocrine glands, metabolism and nutrition	8	15.09	97	6.26	0.927
Mental and behavioral perturbations	3	5.66	83	5.33	0.975
Ophthalmological diseases	3	5.66	26	1.67	0.920
